# Long-Term Outcomes After Percutaneous Transluminal Pulmonary Angioplasty in Patients With Takayasu Arteritis and Pulmonary Hypertension

**DOI:** 10.3389/fimmu.2022.828863

**Published:** 2022-03-11

**Authors:** Zhiwei Huang, Man Wang, Fenghuan Hu, Xiaoning Liu

**Affiliations:** Fuwai Hospital, National Center for Cardiovascular Diseases, Chinese Academy of Medical Sciences and Peking Union Medical College, Beijing, China

**Keywords:** Takayasu arteritis, pulmonary artery stenosis, pulmonary hypertension, percutaneous transluminal pulmonary angioplasty, reperfusion pulmonary edema

## Abstract

**Objective:**

To investigate the long-term efficacy of percutaneous transluminal pulmonary angioplasty (PTPA) in patients with Takayasu arteritis (TA) and pulmonary artery stenosis and pulmonary hypertension (PH).

**Methods:**

Data from 183 lesions from 79 surgeries performed on 32 patients with TA and PH were analyzed. Symptoms, laboratory investigation results, World Health Organization (WHO) functional class, 6-min walk distance (6 MWD), hemodynamic parameters, and prognosis were analyzed at baseline and follow-up.

**Results:**

The mean (± SD) age of the 32 patients (28 female, 4 male) was 42.8 ± 11.9 years, and the median follow-up was 49.5 months (interquartile range, 26–71 months). Compared with baseline, changes in total bilirubin, N-terminal pro-brain natriuretic peptide (NT-proBNP) level, 6 MWD, and WHO score functional class demonstrated significant differences (*P*<0.001). Echocardiography findings, right and left ventricular diameter, tricuspid annular plane systolic excursion, and estimated pulmonary artery systolic pressure were all improved (*P*=0.016, *P*<0.001, *P*<0.001, *and P*=0.005, respectively). Importantly, repeat right heart catheterization revealed that mean pulmonary artery pressure, pulmonary vascular resistance, and cardiac index also improved significantly at follow-up (*P*<0.001, *P*<0.001, and *P*=0.011, respectively). Pulmonary angiography revealed post-procedure restenosis in 64 (35.0%) lesions underwent PTPA within three to six months. Among three patients who underwent stent implantation, one experienced restenosis. Two patients died during the follow-up period, one from aggravation of right heart failure after lung infection, and the other in a traffic accident.

**Conclusions:**

Results of this study indicated that PTPA significantly improved clinical symptoms, exercise tolerance, and hemodynamic parameters in patients with TA pulmonary artery stenosis and PH. More importantly, reperfusion pulmonary edema significantly decreased, and no patient died of PTPA-related complications with guidance from the pressure wire.

## Highlights

1. Pulmonary artery involvement significantly increased mortality in patients with Takayasu arteritis (TA). Treatment of pulmonary artery stenosis in patients with TA has been mainly limited to medical therapy. Interventional treatment, however, restored pulmonary blood flow and improved clinical symptoms. However, due to the relatively high incidence of intervention-related complications, especially reperfusion pulmonary edema, it is not widely used in clinical practice.
2. Percutaneous transluminal pulmonary angioplasty (PTPA) significantly improved clinical symptoms, exercise tolerance, and hemodynamic parameters in TA patients with pulmonary artery stenosis and pulmonary hypertension (PH).
3. Reperfusion pulmonary edema was significantly decreased and no patient died from PTPA-related complications with guidance of the pressure wire.
4. These results are very likely to change the authors’ previous clinical practice, which was mainly based on medical treatment.

## Introduction

Takayasu arteritis (TA) is a chronic progressive inflammatory disease of unknown etiology that affects the aorta, its major branches, and pulmonary arteries (1). Owing to the lack of specific clinical symptoms, pulmonary artery involvement (PAI) is often overlooked by physicians. The prevalence of pulmonary artery stenosis in patients with TA has been reported to range from 13.3% to 61.7% across different populations (2–4). Pulmonary artery stenosis may lead to pulmonary hypertension (PH), which has a significant impact on patient prognosis (5). Poor outcomes are often attributed to delays in diagnosis. PAI often results in PH, and medication alone is insufficient. Hoffman et al. reported that approximately 40% of all steroid-resistant patients responded to the addition of cytotoxic agents, and approximately 20% of all patients are resistant to any type of treatment (6). Unlike pulmonary artery lesions caused by chronic thromboembolism, surgical treatment of pulmonary artery stenosis associated with arteritis has been avoided. There is a lack of therapeutic experience with endovascular treatment in patients with TA and pulmonary artery stenosis. Data regarding percutaneous transluminal pulmonary angioplasty (PTPA) in the treatment of pulmonary artery stenosis in patients with TA are mainly from case reports or very small series (5, 7, 8) and lack long-term follow-up results. With recent advances in endovascular treatment for chronic thromboembolic PH (9), PTPA has become a promising approach for pulmonary arterial lesions in patients with TA. This study aimed to investigate the long-term efficacy of PTPA in the treatment of pulmonary artery stenosis in patients with TA and PH.

## Methods

### Study Population

Data from 32 consecutive TA patients who underwent pulmonary artery intervention between 2016 and 2021 were included in this study. All patients with TA fulfilled the 1990 American College of Rheumatology criteria for the classification of TA (10). Inclusion criteria were as follows: diagnosis of TA with PAI; PH was defined as a mean pulmonary arterial pressure ≥ 25 mmHg, pulmonary artery wedge pressure ≤ 15 mmHg, and pulmonary vascular resistance >3 Wood units, and confirmed using right heart catheterization (RHC); and underwent PTPA treatment. Exclusion criteria included (patients with) severe chronic kidney disease, with an estimated glomerular filtration rate < 30 ml/min·1.73 m^2^, uncontrolled hypertension (systolic blood pressure ≥ 180 mmHg and/or diastolic blood pressure ≥ 110 mmHg), active infectious disease, and severe cardiac insufficiency that would preclude lying on the treatment table. All participants provided informed written consent and the study protocol was approved by the Institutional Review Board of Fuwai Hospital (Beijing, China; Number: 2020-1399).

### Revascularization Procedure

**Figure 1:**
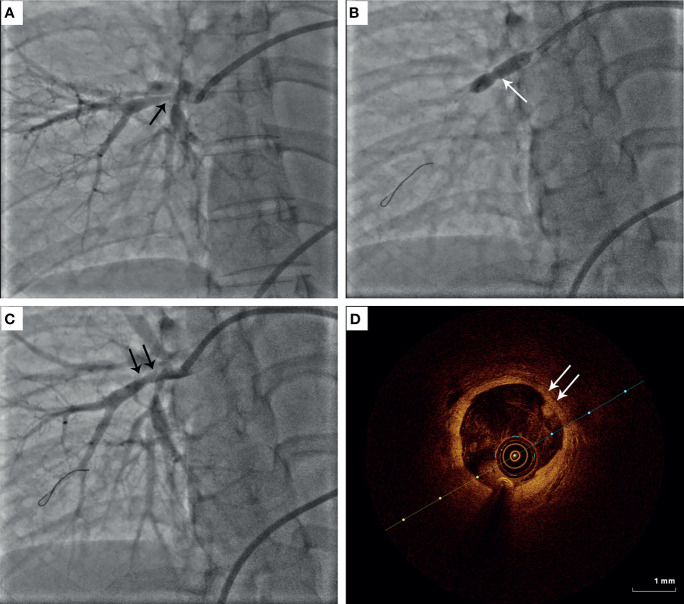
Representative image of percutaneous transluminal pulmonary angioplasty (PTPA) in patients with Takayasu arteritis (TA). **(A)** Diffused stenosis in the right pulmonary artery in TA. **(B)** Balloon inflation at the target lesion of pulmonary artery stenosis. **(C)** Improvement of pulmonary artery stenosis after PTPA. **(D)** Optical coherence tomography findings for pulmonary artery intimal thickness.

Indications for PTPA were follows: pulmonary artery diameter reduction > 70%; mean pulmonary artery pressure (PAP) >30 mmHg and pulmonary vascular resistance (PVR) > 3.75 Wood units (300 dyne • s/cm^-5^); World Health Organization (WHO) functional class ≥2; normal erythrocyte sedimentation rate (ESR) and C-reactive protein (CRP) level. According to the criteria proposed by Kerr (11), all patients must have reached clinical remission before the procedure. All participants were administered aspirin (100 mg/day) and clopidogrel (75 mg/day) for at least five days before the intervention. PTPA was performed *via* femoral vein access with local anesthesia. An 8 F introducer sheath was inserted using the Seldinger technique. An 8 F guide catheter (MPA1or JR4) was then inserted through the sheath, and a 0.014-inch guidewire was passed through the target lesion. Unfractionated heparin was administered at a dose of 1 mg/kg to maintain the appropriate activated clotting time. Patients underwent RHC before and after PTPA and at follow-up examinations. Pulmonary artery angiography was performed before each PTCA to select and identify the target lesions but not after the intervention. Balloon size was determined by measuring vessel diameter using an imaging ruler and, occasionally, optical coherence tomography such as **Figure 1**. Based on the diameter of the normal vascular segment, which included the targeted lesions, a relatively small balloon was used for balloon expansion, and then the balloon diameter was sequentially increased to obtain a larger vascular lumen and greater pulmonary artery blood flow. According to a previous study (9), balloon dilation was stopped to avoid reperfusion pulmonary edema (RPE) when the distal mean PAP, indicated by the pressure wire after each dilation, reached 35 mmHg. Successful dilation was defined as a pressure ratio of distal to proximal pressures across the target lesion, as detected by the pressure wire, of ≥ 0.8. Furthermore, for patients with multiple lesions, a scheme involving multiple procedures was adopted. Additionally, before PTPA, most patients underwent targeted therapy for PH such as bosentan, ambrisentan, sildenafil, tadalafil, or beraprost. After the operation, patients who received PTPA alone continued to take aspirin and clopidogrel for 1 month; for those who underwent stent implantation, aspirin and clopidogrel were used for 3–6 months. Glucocorticoids and/or immunosuppressive agents were administered preoperatively and at follow-up.

### Follow-Up

Follow-up visits were scheduled at 6 and 12 months, and each year after discharge. Routine blood tests, liver function, renal function, N-terminal pro-B-type natriuretic peptide (NT-proBNP), CRP level, ESR, electrocardiogram, echocardiogram, WHO functional class, 6 MWD, and medications were monitored. The patients were recommended to undergo RHC at 12 months postoperatively.

### Statistical Analysis

Continuous variables with a normal distribution are expressed as mean ± standard deviation (SD), and continuous variables without normal distribution are expressed as median (interquartile range [IQR]). Categorical variables are expressed as absolute number and percentage. The paired *t*-test or Mann-Whitney U test was used to compare differences between groups for continuous variables, and categorical variables were compared using the chi-squared (χ^2^) test between baseline and postprocedural assessments. Differences with P < 0.05 were considered to.be statistically significant. Statistical analyses were performed using SPSS version 21 (IBM Corporation, Armonk, NY, USA).

## Results

### Characteristics of TA Patients With PH

A total of 32 TA patients (28 female, 4 male [ratio 7:1]; mean [± SD] age, 42.8 ± 11.9 years) with PH who underwent PTPA were included in this study. Four patients exhibited renal artery stenosis resulting in secondary hypertension: subclavian artery stenosis, n = 1; carotid artery stenosis, n =1; and PAI, n = 2. WHO functional class among the 32 patients was distributed as follows: class II, n = 15 (46.9%); class III, n =16 (50.0%); and class IV, n = 1 (3.1%). Baseline clinical characteristics of the enrolled patients are summarized in **Table 1**.

**Table 1:** Anthropometric and clinical characteristics in TA patients with PH at baseline.

Variables	TA with PH (n = 32)
Clinical characteristics	
Age, years	42.8 ± 11.9
Disease duration at the first procedure, months	48 (36, 108)
Follow up, months	49.5 (41, 62)
Female, n (%)	28 (87.5%)
WHO FC I-II	15 (46.9%)
WHO FC III-IV	17 (53.1%)
Comorbidities, n (%)	
Secondary hypertension	4 (12.5%)
Dyslipidemia	1 (3.1%)
Diabetes mellitus	0 (0.0%)
CAD	0 (0.0%)
PAD	6 (18.8%)
Smoking	0 (0.0%)
Medications, n (%)	
Prednisone	24 (75.0%)
Immunosuppressants	3 (9.4%)
PH-targeted agents	30 (93.8%)
PDE5 inhibitors	23 (71.9%)
ERA	21 (65.6%)
Prostacyclin Analogue	7 (21.9%)

Data are presented as the means ± SD, Median or as numbers and percentages; TA, Takayasu’s arteritis; CAD, coronary artery disease; PAD, peripheral arterial disease; PDE5, phosphodiesterase 5; ERA, endothelin receptor antagonist; PH, pulmonary hypertension.

### Procedural Features

PTPA was successfully performed for 183 lesions from 79 operations in 32 patients. Among these, 180 lesions were treated with PTPA alone, and 3 were treated with stent placement. Two (6.3%) patients developed RPE during the postoperative period, which was relieved after administration of furosemide, dexamethasone, and noninvasive ventilator adjuvant therapy. Three (9.4%) patients underwent vascular dissection without extravascular leak(s). Dissected arteries were repeatedly compressed using a suitable balloon at 4–6 mmHg pressure for 3–5 min until the absence of apparent contrast agent retention.

### Follow-Up

**Figure 2:**
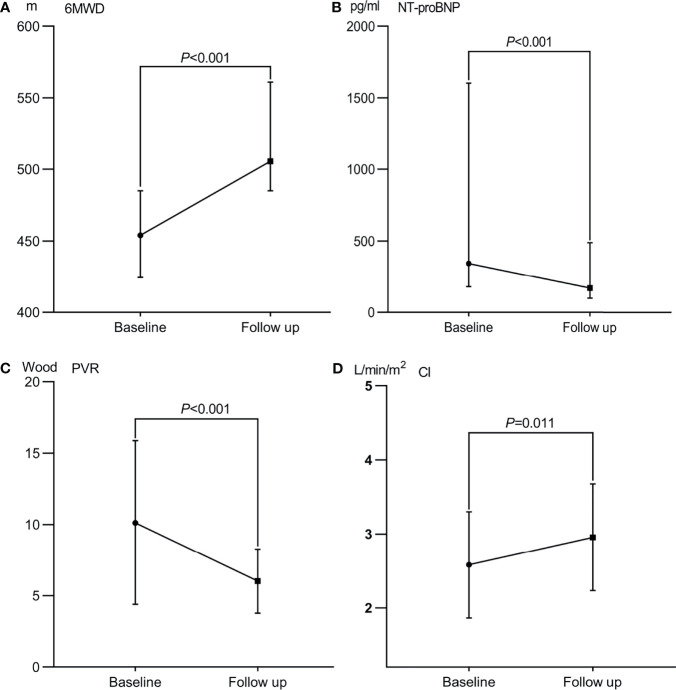
Therapeutic efficacy after percutaneous transluminal angioplasty in patients with Takayasu arteritis and pulmonary hypertension. **(A)** 6 min walk distance (6-MWD). **(B)** N-terminal pro-brain natriuretic peptide (NT-proBNP) level. **(C)** Pulmonary vascular resistance (PVR); **(D)** Cardiac index (CI).

Among the enrolled patients, none were lost to follow-up. The median follow-up was 49.5 months (IQR 26–71 months). After the PTPA procedure, exertional dyspnea was clearly improved in most patients. Pulmonary angiography revealed post-procedure restenosis in 64 (35.0%) lesions underwent PTPA within three to six months. The pressure ratio across the restenotic lesions was measured and, if the ratio was < 0.8, PTPA was repeated. Compared with baseline, changes in total bilirubin, NT-proBNP level, 6 MWD, and WHO functional class were statistically significant (all *P* < 0.001). Echocardiography indicated that right ventricular diameter, left ventricular diameter, tricuspid annular plane systolic excursion (i.e., TAPSE), and estimated pulmonary artery systolic pressure all improved (all *P* < 0.05). Importantly, repeated RHC revealed that mean PAP, PVR, and cardiac index also improved significantly at follow-up (all P < 0.05). These data are presented in **Table 2** and **Figure 2**. Among those who underwent stent implantation, one experienced restenosis. Unfortunately, two patients died during the follow-up period, one from aggravation of right heart failure after lung infection, and the other died due to a traffic accident.

**Table 2:** The changes of percutaneous transluminal pulmonary angioplasty for TA patients with PH.

Variables	Preoperative (n = 32)	Follow up (n = 30)	P
Clinical changes			
WHO functional class, I/II/III/IV	0/15/16/1	12/18/0/0	<0.001
6MWD, m	441.6 ± 59.1	517.0 ± 40.4	<0.001
Blood test			
CRP, mg/l	5.6 ± 7.5	4.8 ± 4.0	0.510
ESR, mm/h	5 (3, 8.5)	6.5 (4.8, 10.0)	0.258
TBIL, mmol/L	19.6 ± 12.2	12.6 ± 6.3	<0.001
NTpro-BNP, pg/mL	344.0 (177.5, 2144.5)	170.5 (98.5, 488.6)	<0.001
ECHO			
RVD, mm	29.5 ± 7.2	27.0 ± 6.7	0.016
LVD, mm	39.0 ± 5.8	42.4 ± 4.5	<0.001
TAPSE, mm	17.2 ± 2.9	19.4 ± 2.6	<0.001
PASP, mm Hg	79.2 ± 26.3	62.0 ± 21.7	0.005
RHC			
Mean PAP, mm Hg	49.7 ± 12.7	37.9 ± 9.6	<0.001
PVR, Wood units	10.1 ± 5.7	6.0 ± 2.3	<0.001
CI, l/min/m	2.58 ± 0.72	2.96 ± 0.72	0.011
PAWP, mm Hg	10.9 ± 1.6	10.7 ± 2.0	0.674

Data are presented as the means ± SD, Median or as numbers and percentages; TA, takayasu’s arteritis; PH, pulmonary hypertension; 6MWD, 6-minute walk distance; CRP, C-reactive protein; ESR, erythrocyte sedimentation rate; TBIL, total bilirubin; RVD, right ventricular diameter; LVD, left ventricular diameter; TAPSE, tricuspid annular plane systolic excursion; PASP, pulmonary artery systolic pressure; RHC, right heart catheterization; PVR, pulmonary vascular resistance; CI, cardiac index; PAWP, pulmonary arterial wedge pressure.

## Discussion

Findings of the present study indicated that PTPA treatment significantly improved clinical symptoms, exercise tolerance, and hemodynamic parameters in TA patients with pulmonary artery stenosis and PH. More importantly, RPE was significantly decreased and no patient died from PTPA-related complications with the guidance of the pressure wire.

Pulmonary artery stenosis in patients with TA is often diagnosed late or misdiagnosed due to nonspecific respiratory manifestations and a lack of symptoms of systemic vessel involvement (12). The prevalence of PAI in patients with TA remains unclear, with varying percentages (13.3%–61.7%) across different studies depending on disease duration, diagnostic criteria, and study population (2–4). Toledano et al. reported a mortality rate of 20.5% in patients with PAI and 33.3% in those with PH (13). Therefore, early diagnosis and timely treatment are critical to improve the prognosis in this particular patient population.

In the past, treatment of TA patients with pulmonary artery stenosis relied mainly on medical therapy with few available surgical options. The main reason is that PTPA can induce RPE as a post-interventional complication, which sometimes leads to significant morbidity and mortality. Feinstein et al. (14) first reported the results of pulmonary balloon angioplasty in a group of 18 patients, in whom 11 (61%) developed RPE after PTPA. In 2012, Kataoka et al. found that 27 of 51 cases (53%) developed RPE, and patients with more severe clinical signs and/or hemodynamic abnormalities at baseline had a higher risk for developing RPE (15). The high incidence of RPE has, however, limited the widespread use of PTPA in clinical practice. In our center, Dong conducted a small-sample study with 14 participants, in which 1 (7.1%) patient died from RPE (8). This is unacceptable because of the high complication rate and mortality.

Fortunately, PTPA had a breakthrough in catheter-interventional therapy in 2014.

Takumi et al. found that the combined approach using pressure wire and PEPSI yielded more efficient clinical results and significantly reduced RPE and vessel complications (9). The key point is that balloon dilation should be stopped to avoid RPE when the distal mean PAP, indicated by the pressure wire after each dilation, reaches 35 mmHg. Successful dilation was confirmed as the pressure ratio of the distal-to-proximal pressures across the target lesion, as detected using a pressure wire, was ≥ 0.8. The results of this study have raised our awareness to this topic and we are encouraged by the findings. We believe that this approach is suitable for the TA patients with pulmonary artery stenosis. Since then, this strategy was adopted in all of our patients undergoing PTPA. Our outcomes demonstrated that PTPA can be performed safely and effectively for TA patients with pulmonary artery stenosis and PH under the guidance of a pressure wire. Two (6.3%) patients experienced RPE during the postoperative period, which was relieved after administration of furosemide, dexamethasone, and noninvasive ventilator adjuvant therapy. None of the patients died from PTPA-related complications. Our results are consistent with a previous report (9) in which the incidence of RPE was 6.9%.

Interestingly, among those who underwent stent implantation, one patient experienced restenosis, although the subjects adopted vigilant inflammatory control during the perioperative follow-up period. A previous report addressing interventional catheterization therapy in TA patients found that biological inflammation at the time of revascularization increased the likelihood of complications in patients with TA by a factor of 7 (16). However, the patient’s CRP and ESR levels were normal during the preoperative and postoperative monitoring. These data suggest that the pathophysiological mechanism of TA involving pulmonary artery stenosis is complex. It is essential, therefore, to explore new biomarkers to monitor disease activity in future studies.

## Limitations

The present study had some limitations, the first of which were data analysis based on a retrospective approach and the inclusion of patients from only a single center. Although prospective enrollment would be ideal, it would be difficult to collect prospective data for such rare diseases. Strengths of our study included the relatively large number of TA patients with pulmonary artery stenosis who underwent pulmonary balloon dilatation and underwent repeated hemodynamic parameters measurement using RHC and long-term follow-up.

## Conclusions

Results of this study indicated that PTPA treatment significantly improved clinical symptoms, exercise tolerance, and hemodynamic parameters in TA patients with pulmonary artery stenosis. More importantly, RPE was significantly decreased, and no patients died from PTPA-related complications with guidance of the pressure wire.

## Data Availability Statement

The raw data supporting the conclusions of this article will be made available by the authors, without undue reservation.

## Ethics Statement

The studies involving human participants were reviewed and approved by Fuwai Hospital, National Center for Cardiovascular Diseases, Chinese Academy of Medical Sciences and Peking Union Medical College. Written informed consent to participate in this study was provided by the participants’ legal guardian/next of kin.

## Author Contributions

XL and ZH designed the study, drafted the manuscript, and ensured the quality of the work. MW and FH supervised the conduct of the study and data collection. ZH drafted the manuscript, and all authors contributed substantially to its revision. All authors contributed to the article and approved the submitted version.

## Funding

This study was supported by the Peking Union Medical College Youth Fund.

## Conflict of Interest

The authors declare that the research was conducted in the absence of any commercial or financial relationships that could be construed as a potential conflict of interest.

## Publisher’s Note

All claims expressed in this article are solely those of the authors and do not necessarily represent those of their affiliated organizations, or those of the publisher, the editors and the reviewers. Any product that may be evaluated in this article, or claim that may be made by its manufacturer, is not guaranteed or endorsed by the publisher.
